# Emerging phage-encoded endolysins to combat multidrug-resistant *Staphylococcus aureus* to boon prevent surgical site infection

**DOI:** 10.1097/MS9.0000000000002615

**Published:** 2024-10-16

**Authors:** Aruchamy Mohanprasanth, Melaku A. Belete, Muthupandian Saravanan

**Affiliations:** a AMR and Nanotherapeutics Lab, Department of Pharmacology, Saveetha Dental College and Hospital, Saveetha Institute of Medical and Technical Science (SIMATS), Chennai, Tamil Nadu, India; b Department of Medical Laboratory Science, College of Medicine and Health Sciences, Wollo University, Dessie, Ethiopia; c Department of Medical Laboratory Technology, Faculty of Applied Medical Sciences, University of Tabuk, Tabuk, Saudi Arabia

The global rise of multidrug-resistant (MDR) pathogens, notably methicillin-resistant *Staphylococcus aureus* (MRSA), constitutes a serious risk to public health and clinical environments. This bacterial pathogen is notorious for causing a range of infections such as skin and soft tissue infections, alongside pneumonia, and, critically, surgical site infections (SSIs). SSIs impose a significant burden, resulting in longer hospitalizations, higher healthcare expenses, and increased morbidity and mortality rates^1^. Traditional antibiotic therapies are becoming increasingly ineffective against MRSA due to the pathogen’s ability to develop resistance. Consequently, alternative therapeutic approaches are urgently required to address these resilient bacterial infections^2^. One promising approach is the use of phage-encoded endolysins.

Endolysins, also referred to as lysins or murein hydrolases, are enzymes synthesized by bacteriophages (phages) to break down the peptidoglycan layer of bacterial cell walls, allowing the release of phage progeny, demonstrated in Figure 1. Unlike traditional antibiotics, endolysins exhibit a highly specific mechanism of action, targeting essential components of the bacterial cell wall with minimal impact on the surrounding microbiota. This specificity reduces the likelihood of off-target effects and the disruption of beneficial bacterial communities. Additionally, the unique mode of action of endolysins makes it difficult for bacteria to develop resistance, as they aim at structures within the bacterial cell wall that are essential for cell viability and are highly conserved^3^.

**Figure 1:**
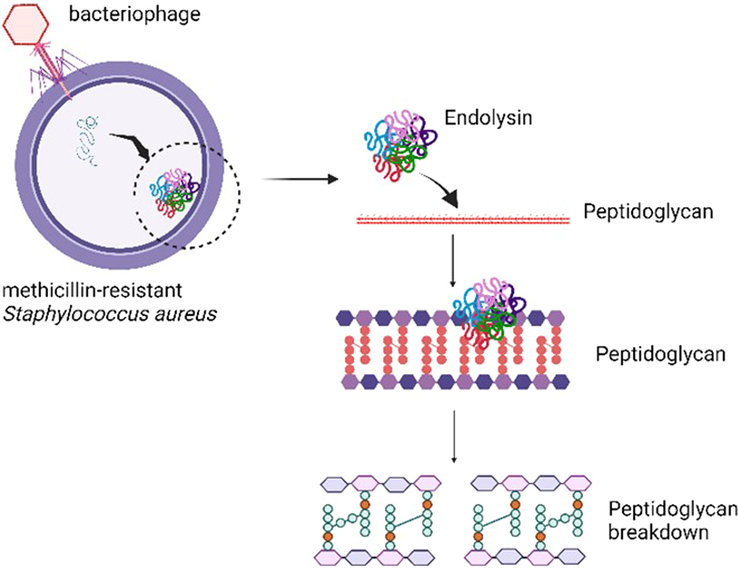
Mechanistic role of endolysin against methicillin-resistant *Staphylococcus aureus*. Endolysin, secreted by bacteriophages, hydrolyzes the cell membrane composed of peptidoglycans. Created with BioRender.com.

Research into phage-encoded endolysins has demonstrated their potent antibacterial activity against a broad spectrum of Gram-positive bacteria, including MDR *Staphylococcus aureus*. These enzymes have shown remarkable efficacy in lysing MRSA cells both *in vitro* and *in vivo*, highlighting their potential as a novel therapeutic option. Studies have shown that endolysins can quickly and effectively eliminate MRSA biofilms, which are challenging to treat with conventional antibiotics due to their protective extracellular matrix and the reduced metabolic activity of embedded bacteria^4^.

The application of endolysins in the prevention and treatment of SSIs is particularly promising. Surgical procedures inherently carry a risk of bacterial contamination, and the presence of MDR pathogens like MRSA exacerbates this risk. Endolysins could be employed prophylactically during surgical interventions to reduce bacterial load and prevent the establishment of infections. For instance, endolysin-based formulations could be applied to surgical wounds or incorporated into surgical dressings, providing a targeted antimicrobial barrier that significantly lowers the incidence of SSIs^5^.

Moreover, endolysins can be engineered for enhanced stability and activity. Protein engineering techniques, such as site-directed mutagenesis and domain shuffling, allow for the optimization of endolysin properties to suit specific clinical applications. This customization can improve the enzyme’s thermal stability, pH tolerance, and binding affinity, thereby increasing its therapeutic potential. Additionally, fusion proteins combining endolysins with other antimicrobial peptides or binding domains can create multifunctional agents with synergistic effects, further boosting their antibacterial efficacy^6^.

Another advantage of endolysins is their rapid bactericidal action. Unlike antibiotics that may require hours to days to achieve therapeutic concentrations and effect, endolysins can lyse bacterial cells within minutes of contact. This rapid action is particularly advantageous in a surgical setting where immediate reduction of bacterial load is critical to preventing infection. Additionally, the ability of endolysins to disrupt biofilms and target dormant bacteria addresses one of the major challenges in treating SSIs caused by biofilm-forming pathogens^7^.

The development and clinical implementation of endolysin-based therapies do face challenges, including regulatory hurdles and the need for extensive clinical trials to establish safety and efficacy. However, the growing body of evidence supporting the antibacterial potency and specificity of endolysins provides a strong foundation for their potential use in combating MDR pathogens. As research progresses, it is anticipated that endolysin-based treatments will be integrated into the antimicrobial arsenal, offering a much-needed solution to the escalating problem of antibiotic resistance^7,8^.

In conclusion, phage-encoded endolysins represent a promising and innovative approach to combating multidrug-resistant *S. aureus*, particularly in the prevention and treatment of surgical site infections. Their unique mechanism of action, specificity, and rapid bactericidal activity make them ideal candidates for developing new antimicrobial therapies. As the prevalence of MDR pathogens continues to rise, the exploration and application of endolysins could provide a critical breakthrough in reducing the burden of SSIs and improving patient outcomes in surgical settings. Continued research and investment in this field are essential to fully realize the potential of endolysins and to bring these powerful enzymes from the laboratory to clinical practice.

## Ethical approval

Ethics approval was not required for this editorial.

## Consent

Informed consent was not required for this editorial.

## Source of funding

No source of funding.

## Author contribution

A.M.: conceptualization, investigation, and writing – original draft preparation; M.S.: conceptualization, investigation, writing – reviewing and editing, and supervision; M.A.B.: conceptualization, writing – reviewing and editing, and supervision.

## Conflicts of interest disclosure

The authors declare no conflicts of interest.

## Research registration unique identifying number (UIN)

Not applicable.

## Guarantor

Melaku Ashagrie Belete.

## Data availability statement

Not applicable.

## Provenance and peer review

Not applicable.
